# WCNN3D: Wavelet Convolutional Neural Network-Based 3D Object Detection for Autonomous Driving

**DOI:** 10.3390/s22187010

**Published:** 2022-09-16

**Authors:** Simegnew Yihunie Alaba, John E. Ball

**Affiliations:** Department of Electrical and Computer Engineering, James Worth Bagley College of Engineering, Mississippi State University, Starkville, MS 39762, USA

**Keywords:** autonomous driving, deep learning, LIDAR data, wavelets, 3D object detection

## Abstract

Three-dimensional object detection is crucial for autonomous driving to understand the driving environment. Since the pooling operation causes information loss in the standard CNN, we designed a wavelet-multiresolution-analysis-based 3D object detection network without a pooling operation. Additionally, instead of using a single filter like the standard convolution, we used the lower-frequency and higher-frequency coefficients as a filter. These filters capture more relevant parts than a single filter, enlarging the receptive field. The model comprises a discrete wavelet transform (DWT) and an inverse wavelet transform (IWT) with skip connections to encourage feature reuse for contrasting and expanding layers. The IWT enriches the feature representation by fully recovering the lost details during the downsampling operation. Element-wise summation was used for the skip connections to decrease the computational burden. We trained the model for the Haar and Daubechies (Db4) wavelets. The two-level wavelet decomposition result shows that we can build a lightweight model without losing significant performance. The experimental results on KITTI’s BEV and 3D evaluation benchmark show that our model outperforms the PointPillars-based model by up to 14% while reducing the number of trainable parameters.

## 1. Introduction

LiDAR 3D object detection in robotics applications, such as autonomous vehicles (AVs), is essential for detecting other vehicles, pedestrians, and cyclists. Technology development companies such as Waymo [1] and car manufacturing companies such as Bosch [2] have tested vehicles equipped with different sensors, including LiDAR. Because of the rapid growth of deep learning (DL) methods in computer vision, research on 3D object detection has increased. Due to this growth, the performance of DL methods has improved. Among these, CNNs have shown a significant performance improvement and state-of-the-art performance in several computer vision tasks, such as image classification and 2D object detection. Although the pooling operation reduces the size of the features at every layer for strides of more than one, it causes information loss, which can affect the detection performance.

Another limitation of CNN is the lack of interpretability. A CNN is a universal function approximator. However, it is considered a black-box model because of nonlinear mapping and unclear working mechanisms [3]. This characteristic makes it difficult to understand how CNN learns and interacts with other models. Thus, it is hard to understand the soundness and weaknesses of the model and diagnose, as well as correct, potential problems [3]. Because of these problems, model development often relies on trial and error [4].

The discrete wavelet transform (DWT) can also enlarge the receptive field by considering more spatial context and employing multilevel decomposition filters (using low-frequency and high-frequency filters at each level). We can also enlarge the receptive field by using filters of larger sizes, but this increases the computational cost. Dilated convolution is implemented to enlarge the receptive field [5]. However, dilated convolution suffers from a gridding effect [6] because of the combination of pixels from different neighbors.

In the signal-processing community, it is common to transform the spatial signal into a frequency domain representation, such as using a Fourier transform to use the dual property of convolution in the spatial domain and element-wise multiplication in the frequency domain. Rippel et al. [7] proposed a Fourier-transform-based convolutional neural network to use the dual property and tested it on the ImageNet [8] dataset. However, this is analogous to the conventional CNN in the frequency domain. The wavelet transform helps to keep both the spatial and frequency information. In this paper, we built a multiresolution analysis in the neural network with skip connections, WCNN3D: **W**avelet **C**onvolutional-**N**eural-**N**etwork-Based **3D** Object Detection, to mitigate the information loss due to the pooling operation. The standard 2D convolution operates in the spatial domain, which drops the high-frequency component. Designing wavelet-based CNN helps use high-frequency components essential for learning and classifying images or other data. In a DWT, only the low-frequency component is decomposed into low-frequency and high-frequency components at each level of decomposition. However, we kept the high-frequency component in each multilevel DWT decomposition by concatenating the lower-frequency and higher-frequency components to enrich the features at each level of decomposition.

The point cloud data were encoded into point pillars to make the dataset suitable for 2D convolution and were input for the backbone and detection modules. PointPillars uses pointNets to learn a representation of point clouds in a vertical column (pillars) [9] instead of using fixed encoders. We can apply 2D convolution to the encoded pointpillar data, which reduces the computation burden of 3D convolutions. The rest of the paper is structured as follows. Section 2 presents related work. The background information and intuition of wavelets are summarized in Section 3. Section 4 summarizes the proposed model architecture and subnetworks, such as feature extraction and detection head, loss functions, and data augmentation techniques. Results and analyses of the experiment are presented in Section 5. The last Section 6 concludes the work.

## 2. Related Work

This section summarizes 3D object detection using camera images and LiDAR point clouds.

### 2.1. 3D Object Detection Using Camera Images

Camera sensors are rich in texture and color information but lack depth information. Thus, 3D object detection using the camera is challenging. Different methods have been proposed to solve the lack of depth and minimize the performance drop. Some methods use pseudo-LiDAR techniques, whereas others use stereo images and geometric constraints to solve the lack of depth in images [10]. The pseudo-LiDAR methods predict the depth of each image pixel and generate pseudo-LiDAR representation, such as [11,12]. The stereo-image-based methods use stereo images to predict depth information and further generate 3D bounding box information, such as [13,14]. On the other hand, geometric-based methods use different geometric constraints, such as ground planes and the object shape [15,16], to estimate depth information.

Although different techniques have been proposed to solve the camera’s lack of depth information, the performance of the proposed models is lower than 3D sensors such as LiDAR. For example, the pseudo-LiDAR data do not perform similarly to LiDAR data because of the image-to-LiDAR generation error [10].

### 2.2. 3D Object Detection Using LiDAR Point Clouds

Over the last few years, many LiDAR-based 3D object detection methods have been developed. The unstructured and sparse point cloud data are encoded into different representations to learn features: voxels, projections, and raw point cloud methods. VoxelNet [17] transforms point clouds into volumetric (voxel) representations and stacks the volumetric representations to form LiDAR point clouds. SECOND [18] improved voxelNet by introducing sparse convolution [19]. Voxel-FPN [20] also used voxel representation to conduct 3D object detection on point clouds. PointPillars [9] encoded the point cloud data into pillar representation to solve the computational burden of voxel representation due to 3D convolution. Some works, such as PointNet [21] and PointNet++ [22], directly process the raw point cloud for 3D object detection. The raw point cloud implementation has a high computational burden because of the 3D convolutions, besides the sparse and unstructured nature of the point cloud. On the one hand, works such as BirdNet+ [23] projected the LiDAR point cloud into bird’s-eye view (BEV) and applied 2D standard convolution on the projected data. Some methods use more than one sensor to solve the limitation of individual sensors and use the best out of each sensor. MV3D [24] and AVOD [25] fuse the projected BEV data with image data to enhance the 3D object detection performance. Another technique is integrating more than one LiDAR data representation, such as voxel and raw point cloud. PV-RCNN [26] integrated the voxel representation and the raw point cloud to obtain the best of the two representations. Although the model showed a large margin performance improvement over the PointPillars [9] network, the model’s parameter is larger due to the 3D convolutions.

Many works adopt the pillar representation, such as [27,28,29], because of its ability to learn feature representation instead of extracting features using fixed encoders like VoxelNet [17]. Additionally, pillar representation is fast because we can apply 2D convolutions to the extracted features instead of 3D convolutions. Caine et al. [27] proposed a pseudo-labeling domain adaptation method for a 3D object detection student–teacher network. Zhou et al. [29] presented a multiview fusion 3D object detection network from vertical column pillars and a perspective view. In this work, besides using the pillar encoding of point pillars to reduce the computational burden, we designed a new wavelet-based convolutional neural network for 3D object detection to minimize the information loss because of the pooling operation in the standard 2D convolution and to enlarge the receptive field.

Wavelet decomposition is well known and widely used in signal processing, but its usage in the computer vision community is limited. Works such as [30,31,32] embed wavelets into the neural network to carry out 2D classification, image segmentation, and image restoration. Fujieda and Takayama [30] presented a multi-resolution analysis and CNNs combination for texture classification and image annotation. The authors achieved a significant performance improvement over the CNN models with fewer parameters. Shen proposed WaveSNet [31], a wavelet-integrated deep network for image segmentation applications. The author integrated discrete wavelet transform into U-Net [33], SegNet [34], and DeepLabv3+ [35] models for effective image segmentation. Liu et al. [32] proposed a multi-level wavelet convolutional neural network (MWCNN) for image denoising, single image super-resolution, JPEG image artifacts removal, and classification. Even though the wavelet has shown significant performance in the above works and other signal processing tasks, it is not common in object detection, especially in 3D object detection. Three-dimensional object detection networks need to fulfill criteria such as being lightweight to run on real-time speed and accurate to give error-free information [36]. We introduce the first wavelet-based lightweight 3D object detection model for autonomous driving. This paper uses the term wavelet and discrete wavelet transforms interchangeably. We focus only on discrete wavelet transforms in this work, so both terms refer to the discrete wavelet transform.

### 2.3. Contributions

We designed a neural network by adding a high-frequency component to the standard spatial domain of CNN to enrich the feature representation. Additionally, we used the DWT as a downsampling operator to avoid information loss because of the pooling operation and to enlarge the receptive field using the multiresolution filters. The main contributions of the paper can be summarized as follows.

1. To the best of our knowledge, our wavelet-based convolutional neural network model that incorporates the high-frequency component into the spatial domain to enhance the performance is the first work in 3D object detection.
2. The model has high-frequency and low-frequency filters covering a large spatial domain to enlarge the receptive field, which improves the performance of the models.
3. We removed the standard pooling operation of CNN to avoid information loss and to design wavelet-based CNN without pooling using the subsampling operation. The biorthogonal property of wavelets helps us to perform subsampling without information loss. We applied IWT to enrich the feature maps for the detection head and fully recover the lost detail information.

We show the performance of the model on the KITTI dataset. The model significantly improves the performance over the standard convolutional neural network models.

## 3. Wavelet-Based Convolutional Neural Network

Spectral approaches have been studied well in the signal and image processing community and have achieved significant results, such as [31,37,38]. To use the advantage of wavelets, we designed a multiresolution [39] wavelet-analysis-based CNN for 3D object detection. The multiresolution analysis property of wavelets is advantageous in detecting objects of different scales.

**Figure 1:**
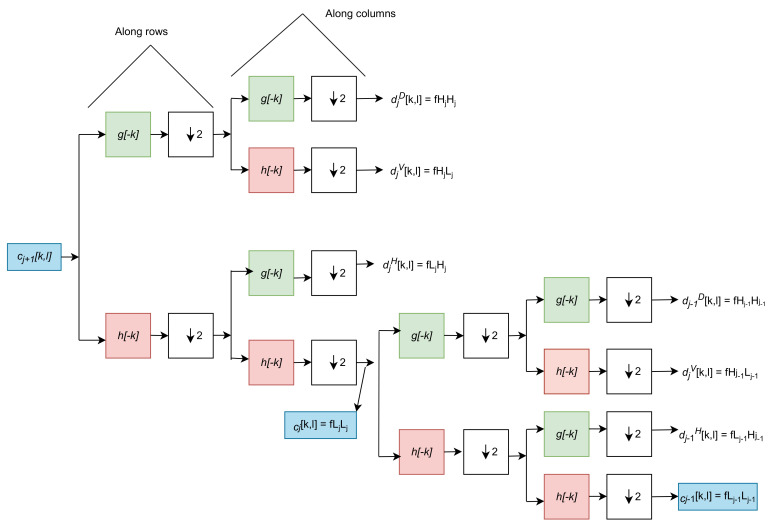
Two−stage 2D wavelet decomposition. The lower-frequency filter coefficient (approximation coefficient) decomposes into lower and higher filter coefficients at each stage of the wavelet decomposition. The decomposition starts with rows downsampling by a factor of two, followed by the same operation along with the columns (the operation can be carried out vice versa, starting with a column and then a row), where *c* is the low-frequency coefficient (coarse coefficient), *d* is the high-frequency coefficient (detailed coefficient), *g* is the high-frequency filter, and *h* is the low-frequency filter. The ↓2 operator is the downsampling/subsampling operator with a factor of two. At every stage of wavelet decomposition, the low-frequency coefficient decomposed into high-frequency and low-frequency coefficients.

The 2D wavelet decomposition on images has approximate (lower-frequency) and detailed (higher-frequency) coefficients. Then, at every stage of decomposition, the lower-frequency component convolves with the image and decomposes further into lower-frequency and higher-frequency coefficients. In a single-stage decomposition, there are four convolutional filters: $f_{LL}$, $f_{LH}$, $f_{HL}$, and $f_{HH}$ as shown in Figure 1. The high frequencies $f_{LH}$, $f_{HL}$, and $f_{HH}$ represent the horizontal, vertical, and diagonal components of the decomposition, respectively. The subscript *L* stands for low-pass filtered and *H* for high-pass filtered. The approximation information is represented by $f_{LL}$, which contains the maximum information of the decomposition. For example, convolving an image ***x*** with the four convolutional filters decomposes the image into sub-band images of $x_{LL}$, $x_{LH}$, $x_{HL}$, and $x_{HH}$. The convolutional filters differ based on the wavelet, such as a Haar or a Daubechies wavelet. For example, for the Haar wavelet, the filter coefficients are defined as:

$$f_{LL}=1/2\left[\begin{array}{cc} 1 & 1\\ 1 & 1 \end{array}\right],f_{LH}=1/2\left[\begin{array}{cc} -1 & -1\\ 1 & 1 \end{array}\right],f_{HL}=1/2\left[\begin{array}{cc} -1 & 1\\ -1 & 1 \end{array}\right],f_{HH}=1/2\left[\begin{array}{cc} 1 & -1\\ -1 & 1 \end{array}\right].$$

The operation of DWT for sub-band images can be expressed as $x_{LL}$ =($f_{LL}$ ∗ ***x***)↓2, $X_{LH}$ = ($f_{LH}$ ∗ ***x***)↓2, $x_{HL}$ = ($f_{HL}$ ∗ ***x***)↓2, and $x_{HH}$ = ($f_{HH}$ ∗ ***x***) ↓2, where ∗ is the convolution operator and ↓2 is the downsampling/subsampling operator with a factor of two. Although there is a downsampling operation in the DWT, the original image/data ***x*** are fully recovered using the IWT without information loss due to the biorthogonal property of wavelets. In the standard convolution, deconvolution and max-unpooling operations are commonly used to increase the resolution of the feature map, but these operations cannot recover the lost data details. The IWT increases the resolution of the feature maps and helps to recover the lost data details. Generally, we used the DWT as a downsampling operator and the IWT as an upsampling operator.

The multiresolution analysis of convolution followed by the downsampling operation is equivalent to the standard convolution followed by the pooling operation. For a convolution layer of a standard CNN, for a training set **x** = ($x_{0},x_{1},...,x_{n-1}$) $\in R^{n}$, we can obtain predicted labels **y** = ($y_{0},y_{1},...,y_{n-1}$) $\in R^{n}$.

(1) $$y_{n}=\sum_{i\in N_{n}}w_{i}x_{i}+bias,$$

where *n* is the number of samples, $N_{n}$ is the set of indices of neighbors of $x_{i}$, and $w_{i}$ is the weight. The label *y* can be written using a cross-correlation (i.e., convolution operator in most machine learning usage) ∗ as:

(2) y=x∗w+bias,

where *x* and *w* are vectors.

The convolution operation of the input *x* with weight *w* is analogous to the DWT sub-band image *x* convolving with the filters. The subsampling layer (pooling layer) is applied immediately to the convolution layer to simplify the information in the standard CNN. For example, average pooling with a stride of two reduces the number of outputs by half compared to the corresponding number of inputs. We omitted biases for the next operations to simplify the notation. We can express the generalized form of convolution followed by the pooling operation as:

(3) y=(x∗w)↓p,

where ↓p is downsampling by a factor of *p* and *p* > 1. When *p* = 2, this operation is analogous to the multiresolution analysis of wavelets with lower-frequency and higher-frequency kernels. This operation can be written as:

(4) $$\begin{array}{cc} x_{l} & =(x*f_{l})↓2\\ x_{h} & =(x*f_{h})↓2, \end{array}$$

where $f_{l}$ is the lower-frequency kernel ($f_{LL}$) and $f_{h}$ is the high-frequency kernel, which consists of $f_{LH},f_{HL}$, and $f_{HH}$. Equation (4) can be expressed as:

(5) $$\begin{array}{cc} x_{LL} & =(x*f_{LL})↓2\\ x_{LH} & =(x*f_{LH})↓2\\ x_{HL} & =(x*f_{HL})↓2\\ x_{HH} & =(x*f_{HH})↓2, \end{array}$$

The lower-frequency component is decomposed into hierarchical decomposition repeatedly for several decomposition levels. However, we used high-frequency and low-frequency kernels at each decomposition level to obtain richer information. At a decomposition level *m*, we can generalize the multiresolution analysis as:

(6) $$\begin{array}{cc} x_{l},m+1 & =(x*f_{l},m)↓2\\ x_{h},m+1 & =(x*f_{h},m)↓2, \end{array}$$

The standard CNN discards $x_{h},m$ and uses only the lower-frequency kernel.

(7) $$x_{l},m+1=\left(x*f_{l},m\right)↓2,$$

A single kernel is used in the standard CNN, but we used both low and high-frequency filters as a kernel. Using low and high-frequency filters increases the chance of taking into account more input pixels during the operation, which increases the field of view (receptive field). The receptive field is the size of a region in the input space that a particular feature looks at, which can be described by its central location and size. When the receptive field size increases, more relevant input parts are considered for detection [5]. The receptive field size of single-path networks for each layer can be calculated using the following arithmetic [40]:

(8) $$r_{i}=r_{i-1}+\left(f-1\right)j_{i-1},$$

where $r_{i}$ is the receptive field size for layer *i*, *f* is a filter size of layer *i*, and $j_{i-1}$ is the cumulative stride. The cumulative strides can be updated using $j_{out}=j_{in}*s$, where s is the stride. To understand the intuition of how the DWT increases the receptive field, let us compare the PointPillars [9] backbone and our backbone. All of the filers are 3 × 3 in the PointPillars network; however, we used a combination of low and high-frequency filters. In the DWT, only a low-frequency filter is decomposed in each decomposition layer, but we used a combination of low and high-frequency filters to increase the receptive field size. Each low and high-frequency filter has a size of 2 × 2. When four of the filters, $f_{LL},f_{LH},f_{HL},and\;\;f_{HH}$, are combined, we can obtain 8 × 8 filters, which is larger than the PointPillars filter size.

Equation (8) works only for single-path networks (i.e., a single input to each layer). When multiple inputs to layer/layers exist, the receptive field size should be calculated for each input to the layer. Our network has multiple inputs from previous layers: both the downsampling and upsampling layers. The receptive field size increases when there are multiple inputs [41]. However, the detection /classification accuracy has a logarithmic relationship with the receptive field size [41]. Let us consider only the filter size by keeping all other parameters, such as padding, the same for both PointPillars [9] and our network. From (8), we can see that the receptive field size of our network is greater than the PointPillars network due to the larger filter size (i.e., 8 × 8 is greater than 3 × 3). However, due to the feature reuse design in the downsampling and upsampling layers, our proposed network has multiple inputs, potentially increasing the receptive field size further.

Note that the receptive field is not the only contributing factor to the performance improvement of DL networks. Other factors, such as the number of layers, number of filters per layer, and batch normalization, contribute to the performance of a DL model. Therefore, using a set of kernels with lower-frequency and higher-frequency filters increases the receptive field. Integrating the higher frequency into the standard CNN outperforms the existing 3D object detection models, as shown in the experiment in Section 5.

## 4. WCNN3D Network Architecture and Implementation Details

**Figure 2:**
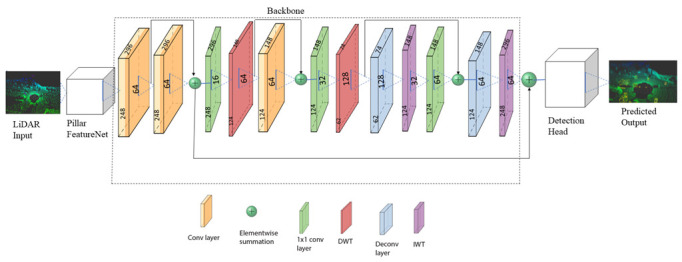
Architecture of the proposed WCNN3D object detection pedestrians and cyclists model for two levels of wavelet decomposition. Each convolutional block comprises three convolutional layers at each level of decomposition. We also used 1×1 convolutions to reduce the channel dimension of feature maps. The raw LiDAR data are encoded through the pillar feature network into a pillar representation. Then, the base network learns features using an expanding and contrasting wavelet convolutional network. The upsampling subnetwork recovers the lost details and increases the feature map of the downsampled features before detection. We did not apply the activation function for the last convolution layer. The upsampled features feed the detection head to predict 3D bounding boxes and classifications of objects. The convolution of down and up layers represent the convolutions of the contrasting and expanding layers, respectively. We also included the feature size (height and width ) and the number of kernels used in each operation. We used 3×3 kernel size, except for 1×1 convolutions, which used a 1×1 kernel size.

The WCNN3D network comprises three major components: the feature network, the base network, and the detection head, as shown in Figure 2. We used the pillar FeatureNet from the PointPillars [9] network to convert the raw point cloud into vertical columns’ pseudo image representation. The base network, our main network, transforms the pillar features into high-level representation. Finally, the detection head predicts classes of objects and regresses 3D bounding boxes for objects.

### 4.1. Pillar FeatureNet

The point cloud data were converted into a pillar representation to avoid the computational burden of 3D convolutions. We used the PointPillar representation from the PointPillars [9] network because it reduces the computational complexity by using 2D convolutions instead of 3D convolutions like VoxelNet [17] while maintaining a high detection performance. The raw point cloud with *x, y, z* and reflectance is discretized into a set of pillars on an evenly spaced *x-y* grid. A pillar is a voxel that has unlimited spatial extent in the *z*-direction [9]. Then, the points in each pillar were decorated with reflectance (*r*), $x{}_{c}$, $y{}_{c}$, $z{}_{c}$, $x{}_{p}$, and $y{}_{p}$. The subscript *c* denotes the distance to the mean of all points in the pillar, whereas the subscript *p* denotes the offset from the pillar *x* and *y* centers. The LiDAR point at this stage is nine-dimensional (i.e., *x, y, z, r,*$x{}_{c}$, $y{}_{c}$, $z{}_{c}$, $x{}_{p}$, and $y{}_{p}$). Most of the pillars are empty due to the sparsity of LiDAR points, so a dense tensor of size (*D, P, N*) was created to reduce the sparsity of the decoded LiDAR point, where *D* denotes the dimension, *P* the number of non-empty pillars per sample, and *N* is the number of points per pillar. This operation reduces memory complexity by focusing on the non-empty pillars only. Applying a linear layer followed by BatchNorm [42] and ReLU [43] generates a tensor of size (*C, P, N*), where C denotes the number of channels. The encoded feature was scattered back to a pseudo-image of size (*C, H, W*), where *H* and *W* denote the height and width, respectively. This representation avoids the computational burden because of 3D convolutions.

### 4.2. Base Network

We designed a simple backbone network based on the DWT and IWT as shown in Figure 2. The network comprises the downsampling and upsampling layers. The downsampling layer performs a 2D DWT, convolution, and ReLU activation. The layer also comprises skip-connections of features from previous layers. The upsampling network was designed using the IWT to increase the feature map resolution and fully recover lost details. The wavelet’s lossless property helps to fully recover the lost information during downsampling, which is essential for the detection head network. We also designed skip-connections between consecutive layers of the downsampling and upsampling layers to enrich feature representation by encouraging feature reuse (knowledge preservation) and mitigating gradient problems for deep networks. An element-wise summation was applied for skip-connections instead of concatenation to decrease the computation burden. We also applied 1×1 convolution to reduce the channel dimensions and maintain the number of trainable parameters to be as small as possible while maintaining a high detection performance. The feature map size and the number of kernels used are given in Figure 2. The input to the first convolution layer is 248 × 296 with 64 kernels. The feature size decreases at each DWT operation and increases at each IWT operation. The batch normalization did not improve the performance, so we opted not to use it for the experiment. This condition might be due to the small number of batch sizes (two in this case because our machine cannot handle more than two batches). All weights were initialized randomly using the uniform distribution as in [44] and PointPillars [9].

### 4.3. Detection Head

We used the single shot detector (SSD) [45] as a detection head followed by regression for 3D bounding box generation. The SSD detection head comprises fully convolutional layers to predict four object predictions for each location (cell). Multiple predictions contain boundary box and confidence scores, which are essential in detecting objects of different scales and aspect ratios. Due to these properties of the SSD, we chose to use the SSD detection head as our object detection head. Then, regression was performed to obtain the 3D bounding box of objects. During the prediction level, non-maximum suppression (NMS) was also applied to suppress duplicate predictions.

### 4.4. Loss

The same loss function as that of VoxelNet [17], SECOND [18], and PointPillars [9], was used. The seven-point bounding box encoding technique (*x, y, z, w, h, l, θ*) was adopted. *x, y*, and *z* are the center of coordinates; *w, l, h* are the width, length, and height, respectively, and θ is the yaw rotation around the *z*-axis. The regression operation between the ground truth and anchors can be defined as:

$$\Delta x=\frac{x^{gt}-x^{a}}{d^{a}},\Delta y=\frac{y^{gt}-y^{a}}{d^{a}},\Delta z=\frac{z^{gt}-z^{a}}{d^{a}}$$

$$\Delta w=\log\frac{w^{gt}}{w^{a}},\Delta h=\log\frac{h^{gt}}{h^{a}},\Delta l=\log\frac{l^{gt}}{l^{a}}$$

$$\Delta \theta =\sin(w^{gt}-w^{a}),$$

where the superscripts *gt* and *a* represent the ground truth and the anchor boxes, respectively. $d^{a}$ = $\sqrt{{\left(w^{a}\right)}^{2}+{\left(l^{a}\right)}^{2}}$ is the diagonal of the anchor box. The SmoothL1 loss and focal loss [46] were used as the localization loss and classification loss, respectively. We used a direction classifier loss, which uses the Softmax loss function, used for angle localization as in SECOND [18] and PointPillars [9]. The SmoothL1 loss can be expressed as:

$$L_{loc}=\sum_{b\in \left(x,y,z,w,l,h,\theta \right)}SmoothL1\left(\Delta b\right),$$

where Δb can be Δx when *b* is *x*, Δy when *b* is *y*, Δϑ when *b* is *θ*, etc.

Similarly, the focal loss can be expressed as follows:

$$L_{cls}=-\alpha_{a}{\left(1-p^{a}\right)}^{\gamma }\log\left(p^{a}\right),$$

where γ is an anchor class probability and $p^{a}$ is an anchor class prediction. For fair comparison, we used the same values as [46] for α = 0.25 and γ = 2. The total loss, which includes the localization loss, classification loss, and directional loss, can be expressed as:

$$L=\frac{1}{N_{pos}}\left(\beta_{loc}L_{loc}+\beta_{cls}L_{cls}+\beta_{dir}L_{dir}\right),$$

where $N_{pos}$ is the number of positive anchors. We used $\beta_{loc}$ = 2, $\beta_{cls}$ = 1, and $\beta_{dir}$ = 0.2, which is the same as [9,46]. Adam optimization with a learning rate of 3 ∗ 10^−4^ with a decay factor of 0.8 for every 15 epochs was used. We trained the model for 160 and 320 epochs with a batch size of two. The model converges faster with fewer epochs too.

### 4.5. Dataset and Data Augmentation

#### 4.5.1. Dataset

We used the KITTI dataset [47], which comprises 7481 training and 7518 testing samples. The model was trained with the LiDAR point clouds that comprise car, pedestrian, and cyclist categories. Each class was evaluated on three difficulty levels: easy, moderate, and hard, based on object size, occlusion, and truncation levels (truncation level refers to what portion of an object is outside the camera view). We divided the original training set into 3712 training samples and 3769 validation samples like [13] and PointPillars [9]. Training the cars with one network and pedestrians and cyclists with another network has become common for 3D object detection [9,17,18,25]. Therefore, we followed the same practice to train cars for one network and pedestrians and cyclists for another network.

We used a maximum number of pillars of 12,000, a maximum number of points per pillar of 100, and a *xy* resolution of 0.16 m. We also trained the model for a maximum of 15,000 pillars and a maximum number of points per pillar of 120. However, increasing the number of points adds a computational burden, so the above values are optimal for performance and computation. At the inference time, NMS was applied to suppress the detection of less than a given IOU value. For the KITTI dataset, cars were evaluated with an IOU of 0.7, whereas pedestrians and cyclists were evaluated with an IOU of 0.5. The *x, y*, and *z* ranges of [(0, 70.4), (−40, 40), (−3, 1)] meters, respectively, were used for cars. A width of 1.6 m, length of 3.9 m, height of 1.5 m, and a *z*-center of −1 m were used for the car’s anchor box. Similarly, the *x, y, z* ranges for pedestrians and cyclists used were [(0, 48), (−20, 20), (−2.5, 0.5)] meters, respectively. A width of 0.6 m, length of 0.8 m, height of 1.73 m, and a *z*-center of −0.6 m were used for the pedestrian anchor box, whereas a width, length, and height of (0.6, 1.76, 1.73) meters, respectively, with a *z*-center of −0.6 m, were used for cyclist anchors. These values are commonly used on the KITTI dataset [47] for different works, such as [9,17,18].

#### 4.5.2. 3D Data Augmentation

Data augmentation has been well-studied in image classification problems compared to object detection models. An appropriate data augmentation, such as color transformation and geometric transformation, improves the generalization of a model. We followed SECOND [18] and PointPillars [9] augmentation techniques. PointPillars created a lookup table for all ground truth 3D bounding box classes. Then, it randomly selected fifteen cars, zero pedestrians, and eight cyclists’ ground truth samples for each sample. However, SECOND [18] used eight, eight, and fifteen ground truth samples for pedestrians, cyclists, and cars, respectively. We used the PointPillars [9] setting for this experiment because of its better performance compared to SECOND [18]. Then, each bounding box was rotated using a uniformly generated rotation angle in the range of [−π/2, π/2].

The local translation was also carried out with samples generated from a normal distribution of zero mean and a standard deviation of [0.25, 0.25, 0.25] for *x, y*, and *z*. Finally, global augmentation was performed to enrich the dataset further. A random mirror flip along the *x*-axis [9,48] was applied, followed by global rotation and scaling [9,17,18]. A global translation was applied that was drawn from a normal distribution with a mean of zero and standard deviation of [0.2, 0.2, 0.2] for *x, y*, and *z*. All experiments were conducted using NVIDIA GeForce RTX 2080 SUPER GPU running on Ubuntu 20.04. Code will be released https://github.com/Simeon340703/WCNN3D accessed on 28 August 2022.

## 5. Results and Discussions

We used the BEV and 3D KITTI datasets as official evaluation metrics for all experiments. Each class’ detection was evaluated based on the three difficulty levels: easy, moderate, and hard. The KITTI dataset’s mean average precision (mAP) was calculated based on the moderate difficulty level evaluation. Table 1 and Table 2 show the experimental results of our model compared with the base model PointPillars [9] and other related state-of-the-art models. Our model outperforms other networks in mAP for both BEV and 3D KITTI dataset evaluation benchmarks. We trained the car’s network for two, three, and four levels of decomposition, but we did not train the pedestrians and cyclists’ network for four levels of decomposition because of the small size of the objects to be decomposed after three levels of decomposition. The three difficult performance levels for cars, pedestrians, and cyclists show that our model’s performance is much higher than other models for both Haar wavelets and Daubechies (Db4) wavelets, except for PV-RCNN for car networks. Although the cars’ performance in the PV-RCNN model, which integrates the voxel form of LiDAR and the raw point cloud, is higher than our model, the pedestrians and cyclists’ network performance is much lower than our model. Additionally, the mAP of our model is higher than PV-RCNN for both BEV and 3D KITTI evaluation metrics. For both the Haar wavelet and Db4 wavelet, the four-level wavelet decomposition gives a higher performance than the three-level and two-level wavelets decomposition, but the drop in the two levels of the wavelet decomposition performance is insignificant compared to the decrease in training parameters. The Db4 performance is higher than the Haar wavelet performance, especially for pedestrians and cyclists.

Embedding the higher frequency into the spatial domain, which contains only the low frequency, enriches the features and helps the detection head for classification and regression tasks. Additionally, the network’s IWT part helps to recover lost details during downsampling. This recovery of lost details helps to increase the performance of the model. The performance improvement ratio of the model on smaller objects, such as pedestrians and cyclists, is greater than the cars’ performance improvement, as shown in Table 1 and Table 2. Therefore, recovering the lost details using IWT is helpful, especially for small objects. Note that most DL models struggle to detect small objects. The moderate and hard difficulty levels of the KITTI dataset decrease the performance of all of the models. However, the detection result of our model even increases at a higher rate for moderate and hard difficulty levels, which proves that adding the higher frequency component helps the model to generalize detection for such conditions.

Even though PV-RCNN improves the performance of the PointPillars by a large margin, the 3D convolution is still the bottleneck and is not a lightweight model for real-time processing. The major challenge of autonomous driving is increasing the performance while keeping the model lightweight. Our car model has 2.64 million, 3.33 million, and 5.6 million trainable parameters for two, three, and four decomposition levels. The pedestrians and cyclists model has 2 million and 2.97 million trainable parameters for two and three decomposition levels, respectively. The only lightweight model is the PointPillars network, with 5.9 million trainable parameters. The PV-RCNN model is not lightweight because of the 3D convolutions used for voxel processing. Therefore, our model is a lightweight model for even real-time processing. Our work will open the door to other researchers in this area to see the object detection network from the other direction to make 3D object detection interpretable, lightweight, and robust for real-time processing.

**Figure 3:**
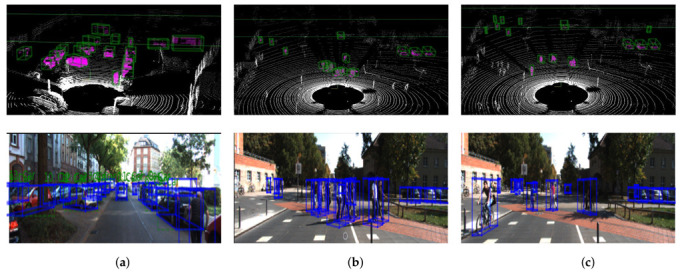
(**a**) Cars, (**b**) pedestrians, (**c**) cyclists. Qualitative analysis of our LiDAR-based 3D object detection model on the KITTI dataset (zoomed view). For better visualization, we used the bird’s eye view (top) and image perspective (bottom). The ground truth boxes are shown in blue, whereas the predicted boxes are in green. Even though the cars (**a**) are overlapped, and some are occluded, the network can detect them.

**Figure 4:**
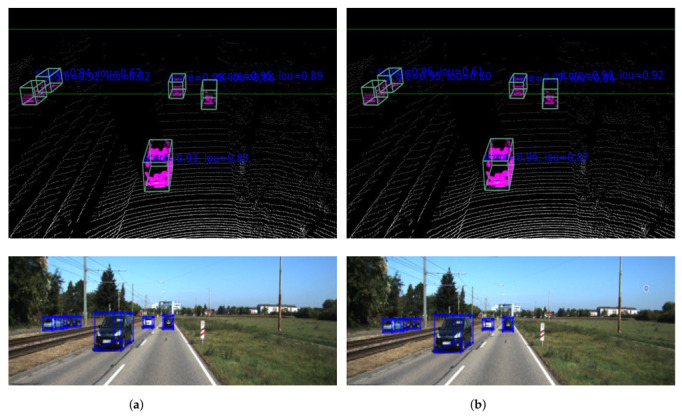
Sample qualitative car class output comparison of the (**a**) PointPillars [9] network and (**b**) our proposed network (zoomed view). For better visualization, we used the bird’s eye view (**top**) and image perspective (**bottom**). The output shows a class score for each image with the corresponding IOU value. The higher the IOU value, the more confident the prediction.

Our model produces an excellent result for cars, pedestrians, and cyclists, as shown in Figure 3. The network can detect strongly overlapped and occluded objects but struggles with a few overlapped, occluded, and faraway objects. Generally, the performance of our network, especially for small objects, such as pedestrians and cyclists, is promising. Figure 4 shows a qualitative comparison of the PointPillars [9] network and our proposed network. It is challenging to differentiate the output of different networks with visualization unless there is a miss detection. However, the images have a confidence score difference for each class. For example, the bottom car in the left and right images has different confidence scores. In the left image (PointPillars network), the bottom car has a 0.93 confidence score and an IOU of 0.83. On the other hand, the same car in the right image has a confidence score of 0.96 with an IOU of 0.87. Other cars in the left and right images also have a different confidence score, as shown in Figure 4.

## 6. Conclusions

In this work, we introduce WCNN3D, a new wavelet-based 3D object detection model for autonomous driving. The experimental results show that removing the pooling operation of CNN and replacing it with wavelet-based CNN increases the detection performance. Designing wavelet-based CNN helps use the high-frequency component of the data thrown away by the standard convolution to enrich features and increase the detection performance of the model. We also used the lower-frequency and high-frequency coefficients of the wavelet as a kernel for the convolution operation, which enlarges the receptive field. The detection result for two levels of wavelet decomposition shows that we can obtain lightweight models without losing much performance while decreasing the number of parameters. The model’s overall performance on the BEV and 3D KITTI evaluation metrics shows that our model outperforms the pillar-based LiDAR 3D object detection models and gives competitive results to sensor fusion-based state-of-the-art models. Therefore, the advantage of wavelet-based CNN is multi-fold. The results of Table 1 and Table 2 show that our model is more suitable for small object detection than other models.

## Figures and Tables

**Table 1:** 3D object detection result for Haar wavelet and Daubechies (Db4) on the KITTI test BEV detection benchmark. Note that ‘2L’ refers to 2-level wavelet decomposition, ‘3L’ refers to 3-level wavelet decomposition, and ‘4L’ refers to 4-level wavelet decomposition. The best results are in bold.

Methods	mAP	Car			Pedestrian			Cyclist		
		Easy	Mod.	Hard	Easy	Mod.	Hard	Easy	Mod.	Hard
MV3D [24]	-	86.62	78.93	69.80	-	-	-	-	-	-
F-pointNet [49]	65.20	91.17	84.67	74.77	57.13	49.57	45.48	77.26	61.37	53.78
PIXOR++ [50]	-	89.38	83.70	77.97	-	-	-	-	-	-
VoxelNet [17]	58.25	89.35	79.26	77.39	46.13	40.74	38.11	66.70	54.76	50.55
SECOND [18]	60.56	88.07	79.37	77.95	55.10	46.27	44.76	73.67	56.04	48.78
PointPillars [9]	66.19	88.35	86.10	79.83	58.66	50.23	47.19	79.19	62.25	56.00
PV-RCNN [26]	70.04	**94.98**	**90.65**	86.14	59.86	50.57	46.74	82.49	68.89	62.41
WCNN3D (Haar-2L) ( ours)	70.99	89.77	87.76	86.32	67.12	62.71	59.04	80.74	62.49	59.28
WCNN3D (Haar-3L) (ours)	71.02	90.05	87.51	86.11	69.20	63.29	59.26	81.54	62.26	58.32
WCNN3D (Haar-4L) (ours)	-	90.02	87.90	86.35	-	-	-	-	-	-
WCNN3D (Db4-2L) (ours)	71.66	90.11	87.83	86.27	**69.48**	**64.52**	**60.07**	**83.69**	62.63	59.45
WCNN3D (Db4-3L) (ours)	71.84	90.12	87.97	**86.46**	68.40	63.20	59.36	82.78	**64.34**	**60.29**
WCNN3D (Db4-4L) (ours)	-	90.20	88.04	86.31	-	-	-	-	-	

**Table 2:** Three-dimensional object detection result for Haar wavelet and Daubechies (Db4) wavelet on the KITTI test 3D detection benchmark. Note that ‘2L’ refers to 2-level wavelet decomposition, ‘3L’ refers to 3-level wavelet decomposition, and ‘4L’ refers to 4-level wavelet decomposition. The best results are in bold.

Methods	mAP	Car			Pedestrian			Cyclist		
		Easy	Mod.	Hard	Easy	Mod.	Hard	Easy	Mod.	Hard
MV3D [24]	-	74.97	63.63	54.0	-	-	-	-	-	-
F-pointNet [49]	56.04	82.19	69.79	60.59	50.53	42.15	38.08	72.27	56.17	49.01
VoxelNet [17]	49.05	77.47	65.11	57.73	39.48	33.69	31.5	61.22	48.36	44.37
SECOND [18]	56.69	83.13	73.66	66.20	41.07	42.56	37.29	70.51	53.85	46.90
PointPillars [9]	59.20	79.05	74.99	68.30	52.08	43.53	41.49	75.78	59.07	52.92
PV-RCNN [26]	62.81	**90.25**	**81.43**	**76.82**	52.17	43.29	40.29	78.60	**63.71**	57.65
WCNN3D (Haar-2L) ( ours)	64.36	87.09	77.39	75.49	58.62	54.57	50.02	79.56	61.13	57.37
WCNN3D (Haar-3L) (ours)	63.16	87.80	77.61	75.71	58.86	53.41	48.94	79.74	58.47	54.71
WCNN3D (Haar-4L) (ours)	-	87.84	77.67	76.00	-	-	-	-	-	-
WCNN3D (Db4-2L) (ours)	65.41	87.75	77.56	75.40	**61.93**	**57.67**	**52.06**	**82.74**	61.01	**57.66**
WCNN3D (Db4-3L) (ours)	64.16	88.57	78.04	76.16	57.88	53.75	49.82	80.14	60.70	56.70
WCNN3D (Db4-4L) (ours)	-	87.83	77.75	75.74	-	-	-	-	-	-

## Data Availability

The KITTI public dataset is available here http://www.cvlibs.net/datasets/kitti/eval_object.php?obj_benchmark=3d (Accessed on 16 February 2022). Code will also be released. https://github.com/Simeon340703/WCNN3D accessed on 28 August 2022.

## Abbreviations

The following abbreviations are used in this manuscript:

CNN	Convolutional Neural Network
DL	Deep Learning
DWT	Discrete Wavelet Transform
IOU	Intersection Over Union
IWT	Inverse Wavelet Transform
mAP	Mean Average Precision
MWCNN	Multi-level Wavelet Convolutional Neural Network
NMS	Non-maximal Suppression
ReLU	Rectified Linear Unit
RCNN	Region-based Convolutional Neural Network
SSD	Single Shot MultiBox Detector
